# The next step to reducing emergency department (ED) crowding: Engaging specialist physicians

**DOI:** 10.1371/journal.pone.0201393

**Published:** 2018-08-20

**Authors:** Jungyeon Kim, Brian J. Yun, Emily L. Aaronson, Haytham M. A. Kaafarani, Pamela Linov, Sandhya K. Rao, Jeffery B. Weilburg, Jarone Lee

**Affiliations:** 1 Department of Global Health and Population, Harvard T.H. Chan School of Public Health, Boston, Massachusetts, United States of America; 2 Department of Emergency Medicine, Massachusetts General Hospital, Boston, Massachusetts, United States of America; 3 Department of Trauma, Emergency Surgery and Surgical Critical Care, Massachusetts General Hospital, Boston, Massachusetts, United States of America; 4 Massachusetts General Physician Organization, Boston, Massachusetts, United States of America; 5 Department of Primary Care, Massachusetts General Hospital, Boston, Massachusetts, United States of America; 6 Department of Psychiatry, Massachusetts General Hospital, Boston, Massachusetts, United States of America; VU university medical center, NETHERLANDS

## Abstract

**Background:**

Much work on reducing ED utilization has focused on primary care practices, but few studies have examined ED visits from patients followed by specialists, especially when the ED visit is related to the specialist’s clinical practice.

**Objective:**

To determine the proportion and characteristics of patients that utilized the ED for specialty-related diagnosis.

**Methods:**

Retrospective, population-based, cohort study was conducted using information from electronic health records and billing database between January 2016 and December 2016. Patients who had seen a specialist during the last five years from the index ED visit date were included. The identification of ED visits attributable to specialists was based on the primary diagnosis of ED visits and the frequency of visit with specialists within a given timeframe.

**Results:**

Approximately 28% of ED visits analyzed were attributable to specialists. ED visits attributed specialists were represented by older patients and occurred more during working hours and early days of week. The most common diagnoses related to ED visits attributed to specialists were Circulatory, Musculoskeletal, Skin, Breast and Mental. Multiple departments, subdivisions and specialists were involved with each ED visit. The number of specialists following the patients who visited the ED ranged from one to six and the number of departments/subdivisions ranged from one to four. Patients that used the ED often were more likely to belong to departments (OR = 1.53) and specialists (OR = 1.18) associated with high ED utilization patterns.

**Conclusion:**

Patients coming to the ED with specialty-related complaints are unique and require full engagement of the specialist and the specialty group. This study offers a new view of connections patients have with their specialists and engaging specialists both at department level and individual specialist level may be an important factor to reduce ED overcrowding.

## Introduction

In 2014 there were 141.4 million emergency department (ED) visits and only 7.9% resulted in hospital admission in the United States [1]. ED crowding is a national problem where 84% of ED visits occurred in metropolitan areas and 18.2% occurred at academic referral hospitals [1–7]. ED crowding leads to adverse health outcomes, poor quality of care and impaired access to care, as well as increases healthcare costs and redundant health service provisions [5,6,8–10].

Much work on reducing ED utilization has focused on primary care practices. Few studies have evaluated ED visits related to specialty practices [11–13]. In 2014, 24% of all ambulatory outpatient visits were visits to medical specialties [1]. Focusing on specialists to reduce ED visits by their patients will only become more important as medical care continues to further sub-specialize. This is especially true at academic referral centers, where a majority of physicians have specialty practices. In order to design successful interventions to reduce ED utilization by patients with ED complaints related to the specialists clinical practice, we need to first understand the problem, patterns and relationships with the ED.

This study aims to examine the use of the ED by discharged ED patients with relationships with specialist physicians, especially if the primary ED diagnosis is attributed to the specialist’s clinical practice. Specifically, our primary outcome was to determine the proportion and characteristics of patients that went to the ED with diagnoses attributed to specialist physicians. Secondary outcomes include: (1) analyzing the complexity of care involved in patients that come to the ED with multiple specialists; (2) determining the risk factors for patients that frequent the ED; (3) categorize the top specialty-related diagnoses of the ED Visits; and (4) determine preventability of the ED visits.

## Materials and methods

### Study design

We conducted a retrospective, population-based, cohort study, using information from electronic health records and billing databases [14] (EPIC Systems Corporation, Verona, WI) on the patients who visited the ED at Massachusetts General Hospital (Boston, MA, USA) between January 1 and December 31, 2016. Massachusetts General Hospital is an urban, academic, quaternary referral center that has 1,011 licensed beds, admits on average 50,000 patients, sees approximately 110,000 ED visits, and 1.5 million outpatient visits annually. The hospital staffs 2,423 physicians and 5,084 registered nurses. For this project, patients that were admitted to the hospital were excluded. We excluded ED visits made by patients who were admitted because we reasoned that patients who discharged, for example, those who were able to remain as outpatients, might have been served by outpatient interventions other than ED visits, such as but not necessarily limited to urgent consultations with their specialists or specialty team. This project was undertaken as a Quality Improvement Initiative at Massachusetts General Hospital, and as such was not formally supervised by the Institutional Review Board per their policies.

### Patients and data processing

To focus on a population of patients that were seen in the ED and discharged home after diagnosis and treatment we included all ED patients that were discharged home during the one-year study period and were attributed to one of our physicians at our hospital. This population represents a subgroup of patients that present to the ED that could potentially be treated in a different setting, such as an office. In order to determine if an ED visit was attributed to a specialist we first determined if the specialist at our hospital was related to the patient who visited ED with the following criteria: (1) one or more visits with specialist during last six months; (2) two or more visits with specialist during last 2.5 years; or (3) five or more visits with at least one in last three years. Next, the specialists were assigned a diagnostic group based on their most frequent billing diagnoses for their outpatient practices using the Clinical Classifications Software (CCS) for International Classification of Diseases (ICD), 9^th^ Revision [15] by Agency for Healthcare research and Quality [16]. We also clustered all primary ED diagnoses using the CCS. Most importantly, if the ED primary diagnoses diagnostic category was the same as the specialist’s assigned diagnostic category, then that ED visit was attributed to that specialist’s clinical practice.

ED visits related to surgical departments and primary care departments were excluded from the study. Seven departments were included in our study:(1) Dermatology; (2) Hematology Oncology; (3) Medicine; (4) Neurology; (5) Pediatrics; (6) Physical Medicine and Rehabilitation; and (7) Psychiatry. The patients records used in our study were fully de-identified before the analysis.

### Measures, outcomes and analysis

First, we examined the relationship between ED visits, patient characteristics and organizational characteristics. Patient characteristics included gender, age and primary diagnosis group. Organizational characteristics included month, day of week and hour when the ED visit was made. We converted age into a categorical variable that consisted of nine categories. A unique ED visit was defined as one visit to any given patient regardless of the number of medical records appeared in the data. If a unique ED visit’s primary diagnosis was attributed to the specialist’s clinical practice, we coded this unique ED visit attributed to the specialist. The one unique ED visit may have multiple numbers of ED records. Based on the ED visit date, time and patient ID, we tagged the unique ED visits and counted only unique ED visits for our analysis. If a unique ED visit had more than one record attributed to a specialist, we coded it as a unique ED visit attributed to that specialist. Our primary outcome variable was a dichotomous variable, that was coded as either “Yes” or “No” in response to whether a given unique ED visit was attributed to a specialist.

Second, to analyze the characteristics of ED visits related to specialists, we analyzed the different characteristics of ED visits attributed to specialists at the level of the departments and subdivisions. We categorized both the Department of Medicine and Department of Pediatrics into eight subdivisions. For the Department of Medicine, the subdivisions included: Allergy-Immunology, Cardiology, Endocrine, Gastroenterology, Infectious Disease, Nephrology, Palliative Care and Pulmonary. For the Department of Pediatrics, the subdivisions included: Pediatric-Cardiology, Pediatric-Endocrine, Pediatric-Gastroenterology, Pediatric-Genetics, Pediatric-Hematology Oncology, Pediatric-Infectious Disease, Pediatric-Pulmonary, and Pediatric-Other.

To analyze the complexity of care coordination of ED visits attributed to specialists, we examined the number of departments, subdivisions and specialists involved with each unique ED visit by diagnosis related group. Because a unique ED visit may involve multiple departments, subdivisions and specialists, we included all the records of ED visits for this analysis.

Based on the distribution of ED visits related to specialists at different levels of medical specialties, we constructed a matrix for ED visits. To map out and explore characteristics of frequent users of ED visits, we defined frequent users based on the median frequency of ED visits. The median frequency of patient in the records was one, that of department in the records was 2,358 and that of frequent specialist was 14. Therefore, we defined a frequent patient when there was more than one unique ED visit, frequent department when there more than 2,358 ED visits and frequent specialist when there was greater than 14 ED visits. We paired frequent patients with frequent specialists if ED visits. This is to see if the frequent patient was attributable to a frequent department or a frequent specialist. We performed multivariate logistic regression to analyze the relationship between frequent patients, frequent departments and frequent specialists.

To classify ED visits that could be potentially treated by specialists not in an ED setting, we applied the New York University’s (NYU) ED algorithm [17] to the primary diagnosis for ED visit. The NYU ED [17] algorithm classified cases into following categories: Non-emergent; Emergent/Primary Care; Emergent-ED care needed-Preventable/Avoidable; Emergent-ED care needed-Not Preventable/Avoidable.

## Results and discussion

### Characteristics of ED visits attributed to specialist physicians

Overall, there were 12,713 unique patients that had 17,553 unique ED visits among patients followed by specialists. Of these visits, there were 3,867 unique patients and 4,861 unique ED visits attributed to specialists. Table 1 shows the overview of characteristics of ED visits between ED visit not attributed and ED visits attributed to specialists. More than a quarter (28%) of ED visits were visits attributed to specialists at our institution (Table 1). Between the two groups, there existed no statistically significant differences in the distribution of gender, month of ED visits and hour of ED visits but there existed significant differences in the distribution of age (P = 0.001), primary diagnosis group (P<0.001), ED visits during working hours (P<0.001), and day of week of ED visit (P = 0.010) (Table 1).

**Table 1:** Characteristics of unique ED visits. (ED visits not attributed to specialists vs. ED visits attributed to specialists).

	ED Visits not attributed to Specialists		ED visits attributed to Specialists		P values
**Total Number of Visits**	12,692	72.31%	4,861	27.69%	
**Patient Characteristics**					
**Gender**	n	%	n	%	0.798
Female	7,011	55.24	2,696	55.46	
Male	5,680	44.75	2,165	44.54	
Unknown	1	0.01	0		
**Age**					
Under 10 years	844	6.65	266	5.47	0.001*
10–19 years	595	4.69	251	5.16	
20–29 years	1,064	8.38	403	8.29	
30–39 years	1,257	9.90	445	9.15	
40–49 years	1,409	11.10	529	10.88	
50–59 years	2,178	17.16	857	17.63	
60–69 years	2,129	16.77	805	16.56	
70–79 years	1,697	13.37	732	15.06	
80 years and over	1,168	9.20	474	9.75	
**Primary Diagnosis Group for ED visit**					<0.001*
Alcohol / Drug, Abuse	178	1.40	138	2.84	
Blood, Immune System	79	0.62	36	0.74	
Burns	30	0.24	0	0.00	
Circulatory	1,441	11.35	935	19.23	
Digestive	1,814	14.29	647	13.31	
Ear, Nose, Mouth, Dental	1,101	8.67	103	2.12	
Endocrine, Metabolic	280	2.21	143	2.94	
Eye	227	1.79	0	0.00	
HIV Infections	2	0.02	0	0.00	
Health Status	516	4.07	66	1.36	
Infections	411	3.24	16	0.33	
Injuries, Poisoning, Comp	661	5.21	17	0.35	
Kidney, Urinary Tract	818	6.45	148	3.04	
Liver, Pancreas	76	0.60	19	0.39	
Mental	249	1.96	493	10.14	
Musculoskeletal	1,705	13.43	689	14.17	
Neoplasm	5	0.04	4	0.08	
Nervous	871	6.86	488	10.04	
Pregnancy, Childbirth	133	1.05	7	0.14	
Reproductive	222	1.75	16	0.33	
Respiratory	816	6.43	375	7.71	
Skin, Breast	1,017	8.01	521	10.72	
Unknown	40	0.32	0	0.00	
**Organization Characteristics**					
**Month**					0.586
Jan	1,321	10.34	476	9.79	
Feb	1,192	9.39	461	9.48	
Mar	1,298	10.23	521	10.72	
Apr	978	7.71	360	7.41	
May	1,051	8.28	396	8.15	
Jun	1,118	8.81	403	8.29	
Jul	1,071	8.44	385	7.92	
Aug	1,025	8.08	423	8.70	
Sep	1,077	8.49	443	9.11	
Oct	988	7.78	362	7.45	
Nov	826	6.51	337	6.93	
Dec	756	5.96	294	6.05	
**Working Hours (9am-5pm Mon-Fri)**					<0.001*
No	8,144	64.17	2,924	60.15	
Yes	4,548	35.83	1,937	39.85	
**Day of Week**					0.010*
Monday	1,888	14.88	779	16.03	
Tuesday	1,789	14.10	761	15.66	
Wednesday	1,846	14.54	715	14.71	
Thursday	1,872	14.75	708	14.56	
Friday	1,819	14.33	668	13.74	
Saturday	1,748	13.77	597	12.28	
Sunday	1,730	13.63	633	13.02	
**Hour**					0.335
0	228	1.80	75	1.54	
1	211	1.66	85	1.75	
2	212	1.67	84	1.73	
3	166	1.31	62	1.28	
4	131	1.03	50	1.03	
5	168	1.32	64	1.32	
6	209	1.65	77	1.58	
7	273	2.15	94	1.93	
8	375	2.95	123	2.53	
9	582	4.59	241	4.96	
10	725	5.71	317	6.52	
11	901	7.10	349	7.18	
12	823	6.48	339	6.97	
13	812	6.40	353	7.26	
14	816	6.43	307	6.32	
15	795	6.26	339	6.97	
16	847	6.67	317	6.52	
17	826	6.51	296	6.09	
18	747	5.89	254	5.23	
19	744	5.86	271	5.57	
20	657	5.18	257	5.29	
21	571	4.50	193	3.97	
22	485	3.82	178	3.66	
23	388	3.06	136	2.80	

*P-value <0.05.

### Unique characteristics of ED visits with diagnoses attributed to specialist physicians

More than 40% of ED visits were made by patients older than 60 years. Of these patients, more of the ED visits were attributed to specialists (41%) than not attributed (39%) (Table 1). The most frequent diagnostic category was digestive (14%) among ED visits not attributed to specialists, while for ED visits attributed to specialists, the most frequent diagnostic category was circulatory (19.23%). The biggest differences in primary diagnosis between the two groups occurred in the mental diagnostic group. The mental diagnosis ranked the fifth most common diagnosis among the ED visits attributed to specialists and accounted for ten percent of ED visits. Conversely, the mental diagnosis ranked 13^th^ among the ED visits not attributed to specialists and accounted for only two percent of ED visits (Table 1). Compared to ED visits not attributed to specialists, more ED visits attributed to specialists occurred during the working hours (40% vs 36%, P<0.001) and earlier in the week (Monday and Tuesday: 32% vs 29%, P = 0.010) (Table 1).

### Complexity of care among patients with specialty-related complaints

**Fig 1:**
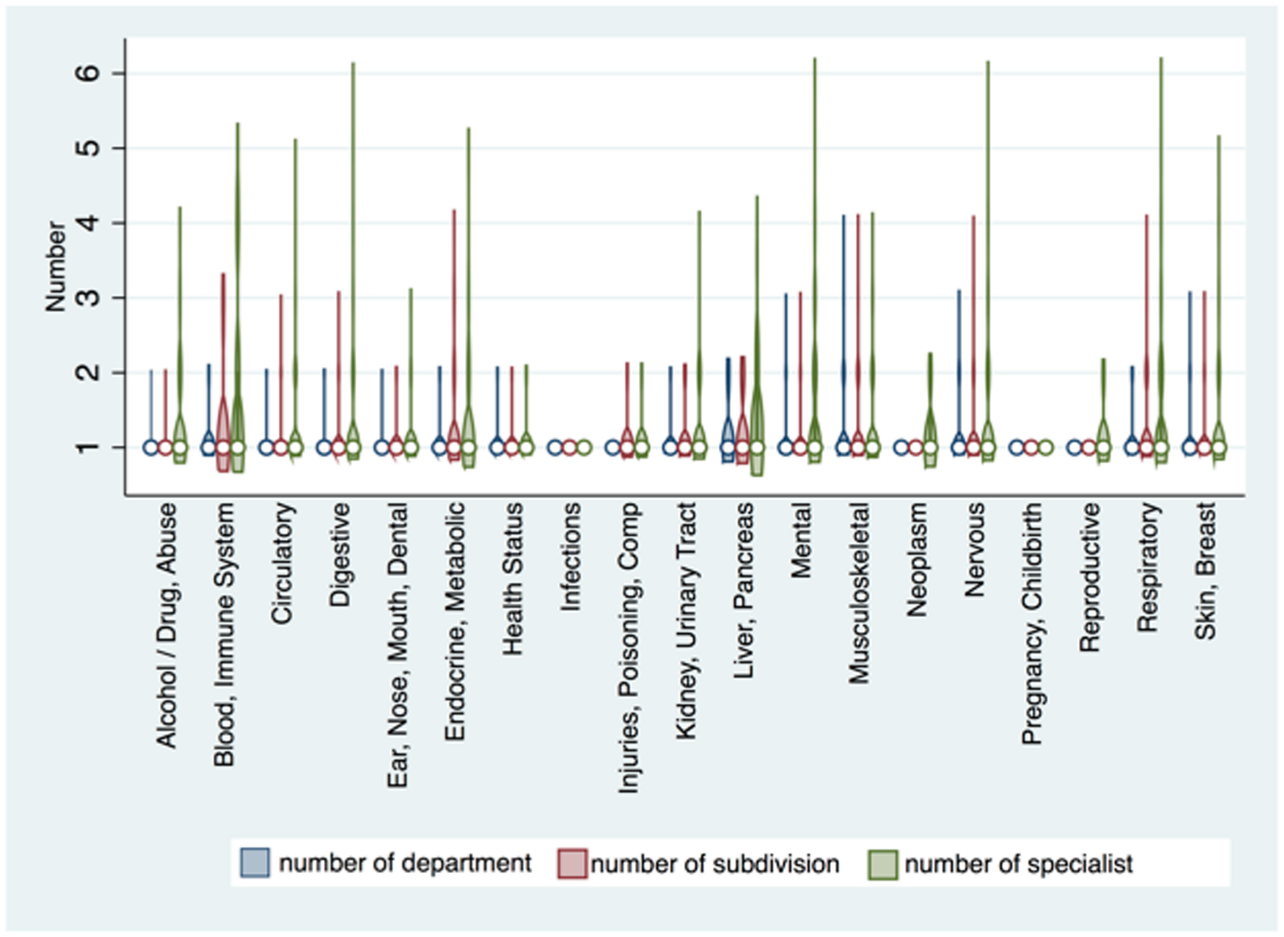
Violin plots for the number of departments, subdivisions and specialists involved per unique ED visits attributed to specialists by diagnosis group.

Fig 1 illustrates the complexity of care by patients that present to the ED. The figure shows the varied distribution of the number of departments, subdivisions and specialists involved per unique ED visits related to specialists across diagnosis groups. Each unique ED visit related to a specialist had a median of one specialists following the patient (Range 1 to 6). Similarly, for each ED visit related to a specialist, the number of departments/subdivisions had a median of one (Range 1 to 4) (Fig 1).

**Fig 2:**
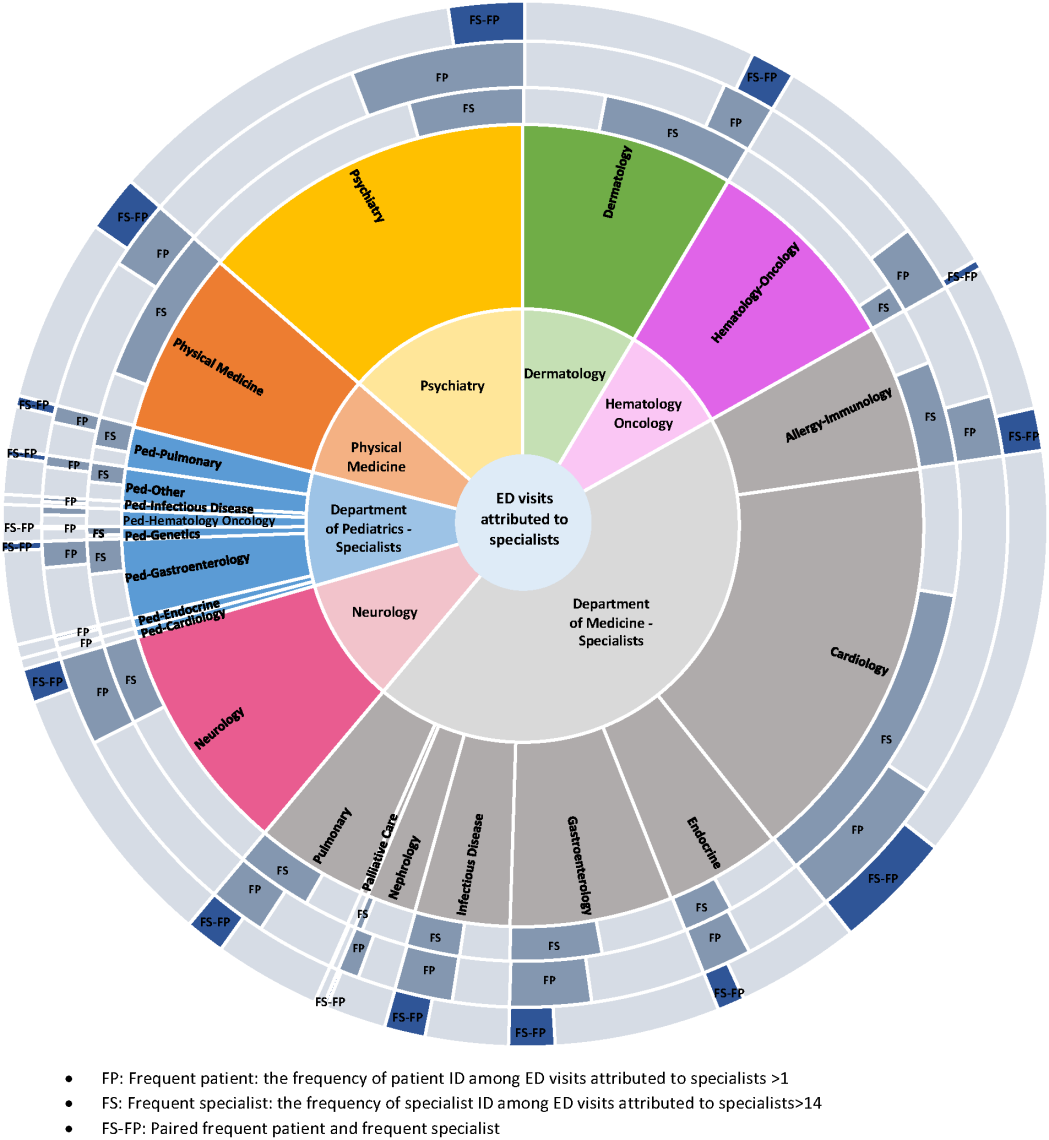
Matrix for ED visits attributed to specialists.

Fig 2 is a matrix that captures hospital-wide characteristics of ED visits attributed to specialists. Fig 2 describes how ED visits attributed to specialists varied over different levels of medical specialty groups. The patterns of ED visits attributed to specialists were distinct across the departments, subdivisions, frequent specialists and frequent patients (Fig 2). For example, the Department of Medicine accounted for approximately 44% of ED visits related to specialists and the subdivision Cardiology accounted for the majority at 37% (1,020). The Department of Hematology-Oncology accounted for 8.29% of total ED visits related to specialists and only 12% of these visits were associated with frequent specialist (Fig 2). Conversely, the Department of Physical Medicine accounted for 7.43% of total ED visits attributed to specialists and 77% of these visits were associated with frequent specialists. The greatest proportion of ED visits attributed to specialists occurred in the Department of Medicine-Specialists, where Cardiology owned the majority (37%) of these visits. Frequent specialists were related to approximately 70% of these Cardiology visits and frequent patients were associated with 30% of these Cardiology visits (Fig 2).

### Risk factors for patients that use the ED frequently

As shown from Table 2, there existed statistically significant relationship between frequent patients, frequent departments (OR = 1.53, P<0.001) and frequent specialists (OR = 1.18, P = 0.002).

**Table 2:** Relationship between frequent patient between frequent department and frequent specialists.

Variable	Unadjusted		Adjusted	
	Odds Ratio (95% CI)	P value	Odds Ratio (95% CI)	P value
Frequent Department	1.54 (1.38–1.72)	<0.001	1.53 (1.37–1.70)	<0.001
Frequent Specialist	1.22 (1.10–1.36)	0.001	1.18 (1.07–1.32)	0.002

The odds of becoming a frequent patient was 53% higher if the patient belonged to a frequent department and was 18% higher if the patient had seen a frequent specialist.

### Top primary diagnoses of patients with specialty-related complaints

For patients who made visits attributed to specialists the top two primary diagnoses were “other chest pain” and “chest pain unspecified”, which were related to the circulatory diagnostic group (Tables 1 and 3). According to the NYU ED algorithm, 39% of “other chest pain” accounted for Emergent-ED care needed—Not Preventable category; while 68% of “chest pain unspecified” accounted for Emergent-ED care needed—Not Preventable category (Table 3). Three diagnoses related to the mental diagnostic group accounted for 10.14% of total diagnoses of visits related to specialists. Specifically, the three diagnoses included “major depressive disorder, single episode”, “unspecified ideations”, and “anxiety disorder unspecified” (Tables 1 and 3). Because the NYU ED algorithm did not classify mental health related or alcohol/drug abuse, diagnoses related to these two ED diagnosis group were unclassified in terms of preventability (Table 3).

**Table 3:** Twenty-five most common diagnosis for ED visits attributed to specialists and New York University ED algorithm [17].

Rank	Diagnosis Description	ICD 10	ED Diagnosis Group	Number of ED visits (n = 4,861)	Pro-portion (%)	Accumu-lated (%)	Emergent			Non-emergent	Psych, Alcohol, Substance abuse, Injury, Unclassified
							ED Care Needed		Primary care treatable		
							Not preventable/avoidable	Preventable/avoidable			
1	OTHER CHEST PAIN	R07.89	Circulatory	180	3.7	3.7	39%	0%	61%	0%	
2	CHEST PAIN UNSPECIFIED	R07.9	Circulatory	159	3.27	6.97	68%	0%	32%	0%	
3	MAJOR DEPRESSIVE DISORDER, SINGLE EPISODE, UNSPECIFIED	F32.9	Mental	123	2.53	9.5	N/A				100% (Psych)
4	HEADACHE	R51	Nervous	111	2.28	11.79	13%	0%	9%	78%	
5	SYNCOPE AND COLLAPSE	R55	Circulatory	93	1.91	13.7	67%	0%	33%	0%	
6	UNSPECIFIED ABDOMINAL PAIN	R10.9	Digestive	90	1.85	15.55	33%	0%	67%	0%	
7	UNS ASTHMA W/ACUTE EXACERBATION	J45.901	Respiratory	73	1.5	17.05	0%	98%	2%	0%	
8	LOW BACK PAIN	M54.5	Musculoskeletal	72	1.48	18.54	11%	0%	15%	74%	
9	COPD WITH ACUTE EXACERBATION	J44.1	Respiratory	71	1.46	20	0%	55%	45%	0%	
10	UNSPECIFIED ATRIAL FIBRILLATION	I48.91	Circulatory	67	1.38	21.37	100%	0%	0%	0%	
11	ALCOHOL DEPENDENCE WITH INTOXICATION, UNSPECIFIED	F10.229	Alcohol / Drug, Abuse	64	1.32	22.69	N/A				100% (Alcohol)
12	ESSENTIAL PRIMARY HYPERTENSION	I10	Circulatory	62	1.28	23.97	0%	19%	16%	56%	8% (Unclassified)
13	EPILEPSY, UNSPECIFIED, NOT INTRACTABLE, WITHOUT STATUS EPILEPTICUS	G40.909	Nervous	61	1.25	25.22	N/A				100% (Unclassified)
14	CONSTIPATION UNSPECIFIED	K59.00	Digestive	57	1.17	26.39	N/A				100% (Unclassified)
15	PALPITATIONS	R00.2	Circulatory	56	1.15	27.55	56%	0%	44%	0%	
16	SUICIDAL IDEATIONS	R45.851	Mental	53	1.09	28.64	N/A				100% (Unclassified)
17	PRECORDIAL PAIN	R06.02	Respiratory	48	0.99	29.62	60%	0%	40%	0%	
18	SHORTNESS OF BREATH	R07.2	Circulatory	48	0.99	30.61	0%	0%	100%	0%	
19	ANXIETY DISORDER UNSPECIFIED	F41.9	Mental	44	0.91	31.52	N/A				100% (Psych)
20	UNSPECIFIED CONVULSIONS	N39.0	Kidney, Urinary Tract	42	0.86	32.38	0%	24%	30%	46%	
21	UTI SITE NOT SPECIFIED	R56.9	Nervous	42	0.86	33.24	0%	75%	25%	0%	
22	RIGHT UPPER QUADRANT PAIN	R10.11	Digestive	39	0.8	34.05	33%	0%	67%	0%	
23	EPIGASTRIC PAIN	R10.13	Digestive	35	0.72	34.77	33%	0%	67%	0%	
24	ALCOHOL ABUSE WITH INTOXICATION, UNSPECIFIED	F10.129	Alcohol / Drug, Abuse	33	0.68	35.45	N/A				100% (Alcohol)
25	PAROXYSMAL ATRIAL FIBRILLATION	I48.0	Circulatory	33	0.68	36.12	100%	0%	0%	0%	

## Discussion

Our study suggests that engagement of specialist is essential to tackling ED crowding especially in urban and academic referral settings. Approximately 28% of ambulatory ED visits were attributable to specialists in our system. Until now interventions to reduce ED crowding have focused on primary care [18–21]. The results of this study suggest that there is an opportunity to develop interventions aimed at reducing ED utilization by focusing on specialty care. This study will extend the spectrum of health service provisions not only non-emergent conditions but also to more complicated and emergent conditions that may not be possible to care for in primary care settings [17].

Additionally, we found that patients that came to the ED with a specialty-related complaint had a higher complexity of care as evidenced by how they are followed by multiple departments, divisions and specialists. This tells us that patients with ED visits with a complaint related to their specialists had a greater number of specialists involved in their care. In other words, if a patient comes to the ED with a specialty-related complaint, their coordination of care is exponentially more difficulty and complex. This leads to increased complexity and difficulty when coordinating the care of patients that present to the ED with specialty-related complaints. For example, ED visits attributed to specialists that belong to some diagnostic groups, such as digestive or respiratory, can require involvement of up to two different departments, four different subdivisions and six separate specialists. Even though these ED visits appear to be a complicated matrix of diagnosis and treatment choice for complex patients, there exists a distinct pattern between frequent ED patients and frequent departments or specialists. Patients that are attributed to frequent departments or specialists are more likely to become frequent ED users. This implies that multi-level engagement both at the departmental level and individual specialist level is necessary to reduce ED utilization by this focused population of patients. The reduction of ED visits by improving ambulatory care is an overarching goal for the accountable care organization (ACO) [22]. To achieve this system-wide goal, it is essential that there is close collaboration between emergency medicine physicians, primary care providers and specialists [22].

ED visits attributed to specialists have different patient characteristics than overall ambulatory ED visits [1]. Patients who visit the ED and had at least one specialist are older. Patients aged 65 years and over accounted for 35% of ED visits attributed to specialists while patients aged 65 years and over accounted for only 15% of the national ED visits [1]. This result is aligned with the findings from a recently published study on cancer-related ED visits [23]. Patients who made ED visits related to cancer were older than patients who made ED visits not related to cancer [23]. Additionally, patients coming to the ED with specialty-related complaints are unique and require full engagement of the specialist and the specialist group. Prior studies have shown that interventions focusing on subspecialties was effective in the reduction of ED utilization. For example, ED visits by patients followed by pediatric gastroenterology, pulmonology, neurology, hematology and infectious disease specialists decreased after engagement and organizational culture change [11,12]. Additionally, the decrease in ED use was associated with a cost savings to ACO in these studies [13].

We also found that engaging specialists could potentially reduce ED visits by approximately 7.3% of ED visits that were emergent-ED care needed-preventable/avoidable and 41.3% of non-emergent visits based on the NYU algorithm [24]. Because NYU algorithm does not classify psych factors, this may underestimate impact of mental health patients. In addition, a recently published study that analyzed ED visit data from the 2006 national hospital Ambulatory Medical Care Survey (NHAMCS) showed that there existed a huge discrepancy between non-emergency complaints of ED visits and ED discharge diagnosis [20]. Although 89% of all ED visits were non-emergent visits based on the chief complaints that were viewed as primary care-treatable conditions, only 6% of all ED visits were primary care-treatable visits based on the discharge diagnoses [20]. This implies that there is an opportunity for specialists to reduce this burden. Future research needs to be done to develop algorithm for specialty care-treatable ED visits.

The diagnoses of mental and nervous were leading reasons followed by circulatory, digestive and skin/breast for ED visits attributed to specialists. This calls for more active engagement of the psychiatric department in designing and implementing interventions to reduce ED use. Mental health boarding remains a serious issue in the ED. Patients presenting with mental health emergencies were found to wait longer for an inpatient bed than non-mental health patients [25].

As the growth rate of mental health emergency related encounters increases, it will be important to focus on population health efforts to reduce ED utilization by providing other appropriate avenues for acute psychiatric care [25].

We also found that approximately 40% of ED visits attributed to specialists were during the working hours and 32% of ED visits attributed to specialists were on Monday or on Tuesday. More urgent access to specialists during the working hours and weekend clinic hours of specialists might help reduce ED visits.

This study has several limitations. Whilst the goal of this research was to achieve a comprehensive picture of ED visits related to specialists to better understand the complexities of care-coordination, the results of this study may not necessarily be generalized to other institutions, such as community hospitals because we included ED patients who had seen specialists in our system. We also did not include patients that were admitted to the hospital, which could potentially increase the pool of patients that could be treated in a different setting. Additional studies at multi-institutions need to be done to more fully examine ED visits related to specialists. Regardless of these limitations, this study provides an overall picture of ED visits related to specialists at a major academic medical center. This study also offers a new view of connections patients have with their specialists and engaging specialists may be an important factor to reduce ED overcrowding.

## Conclusion

Strategies designed to reduce ED crowding requires a paradigm shift to include specialists. While ED visits related to specialists require complex care coordination, engaging specialists may help reduce ED crowding and utilization. This will ultimately lead to the success of an accountable care organization.
